# Artificial Intelligence Uncovers Natural MMP Inhibitor Crocin as a Potential Treatment of Thoracic Aortic Aneurysm and Dissection

**DOI:** 10.3389/fcvm.2022.871486

**Published:** 2022-04-06

**Authors:** Feiran Qi, Yan Liu, Kunlin Zhang, Yanzhenzi Zhang, Ke Xu, Mei Zhou, Huinan Zhao, Shuolin Zhu, Jianxin Chen, Ping Li, Jie Du

**Affiliations:** ^1^Beijing Anzhen Hospital, Capital Medical University, Beijing, China; ^2^Beijing Institute of Heart Lung and Blood Vessel Diseases, Beijing, China; ^3^Beijing Collaborative Innovation Centre for Cardiovascular Disorders, Beijing, China; ^4^The Key Laboratory of Remodeling-Related Cardiovascular Diseases, Ministry of Education, Beijing, China; ^5^School of Life Sciences, Beijing University of Chinese Medicine, Beijing, China; ^6^CAS Key Laboratory of Mental Health, Institute of Psychology, Chinese Academy of Sciences, Beijing, China; ^7^Department of Psychology, University of Chinese Academy of Sciences, Beijing, China; ^8^Department of Physiology and Pathophysiology, School of Basic Medical Sciences, Tianjin Medical University, Tianjin, China

**Keywords:** thoracic aortic aneurysm and dissection, drug therapy, matrix metallopeptidase, crocin, natural compound, artificial intelligence

## Abstract

Thoracic aortic aneurysm and dissection (TAAD) is a lethal cardiovascular condition without effective pharmaceutical therapy. Identifying novel drugs that target the key pathogenetic components is an urgent need. Bioinformatics analysis of pathological studies indicated “extracellular matrix organization” as the most significant functional pathway related to TAAD, in which matrix metallopeptidase (MMP) 2 and MMP9 ranked above other proteases. MMP1-14 were designated as the prototype molecules for docking against PubChem Compound Database using Surflex-Dock, and nine natural compounds were identified. Using a generic MMP activity assay and an aminopropionitrile (BAPN)-induced TAAD mouse model, we identified crocin as an effective MMP inhibitor, suppressing the occurrence and rupture of TAAD. Biolayer interferometry and AI/bioinformatics analyses indicated that crocin may inhibit MMP2 activity by direct binding. Possible binding sites were investigated. Overall, the integration of artificial intelligence and functional experiments identified crocin as an MMP inhibitor with strong therapeutic potential.

## Introduction

Thoracic aortic aneurysm and dissection (TAAD) is an aggressive and progressive aortopathy that, if left untreated, will eventually lead to aortic rupture (1). Patients who suffer aorta ruptures often die from hemorrhagic shock within a few minutes, and the chance of a successful rescue is extremely slim (2). Thus, early intervention for the prevention and treatment of TAAD is particularly important. Since TAAD presents differentially depending on the patient, multiple treatment regimens are available for TAAD. Surgery is the preferred choice for acute type A aortic dissection and aortic aneurysms with a diameter of 55 mm (3), but it cannot prevent recurrence in other parts of the aorta. The recurrence rate of type A aortic dissection after 5 years is 5.3% (4). For uncomplicated Type B dissection, patients can be effectively treated through medical therapy alone. However, the most commonly used drugs in clinical practice treat symptoms by, for example, lowering blood pressure or controlling heart rate (3). There is a lack of drugs to treat or delay the pathological process. In addition, for patients whose aorta has been dilated but the diameter is not large enough for surgery, there is also a lack of drugs that can prevent further aortic dilation. This is especially true in young patients with Marfan syndrome, who will spontaneously develop aortic aneurysms due to genetic mutations (5). Therefore, novel drugs targeting the pathological processes of TAAD for prevention and treatment are urgently needed.

The pathophysiological process of TAAD is characterized by the degradation of the extracellular matrix (ECM) and vascular remodeling, including inflammatory responses, mid-layer elastic fiber breakage, apoptosis of smooth muscle cells, and proteoglycan deposition (6). Several studies have demonstrated that certain targets in these pathological processes, including matrix metalloproteinases (MMPs), type II TGF-β receptor (Tgfbr2), and Ang II receptor 1 (AT1), play important roles in the formation of TAAD and are potential therapeutic targets (7–9). Given that natural products are an important source of drugs that can act on multiple targets simultaneously to produce additive or synergistic effects, we aimed to identify the key targets for intervention in TAAD in a macroscopic and unbiased manner using previous pathological studies and apply artificial intelligence screening to discover new natural therapeutic drugs based on these targets.

## Results

### MMPs Were Identified as Key Proteases of Extracellular Matrix Pathway Related to TAAD

**Figure 1:**
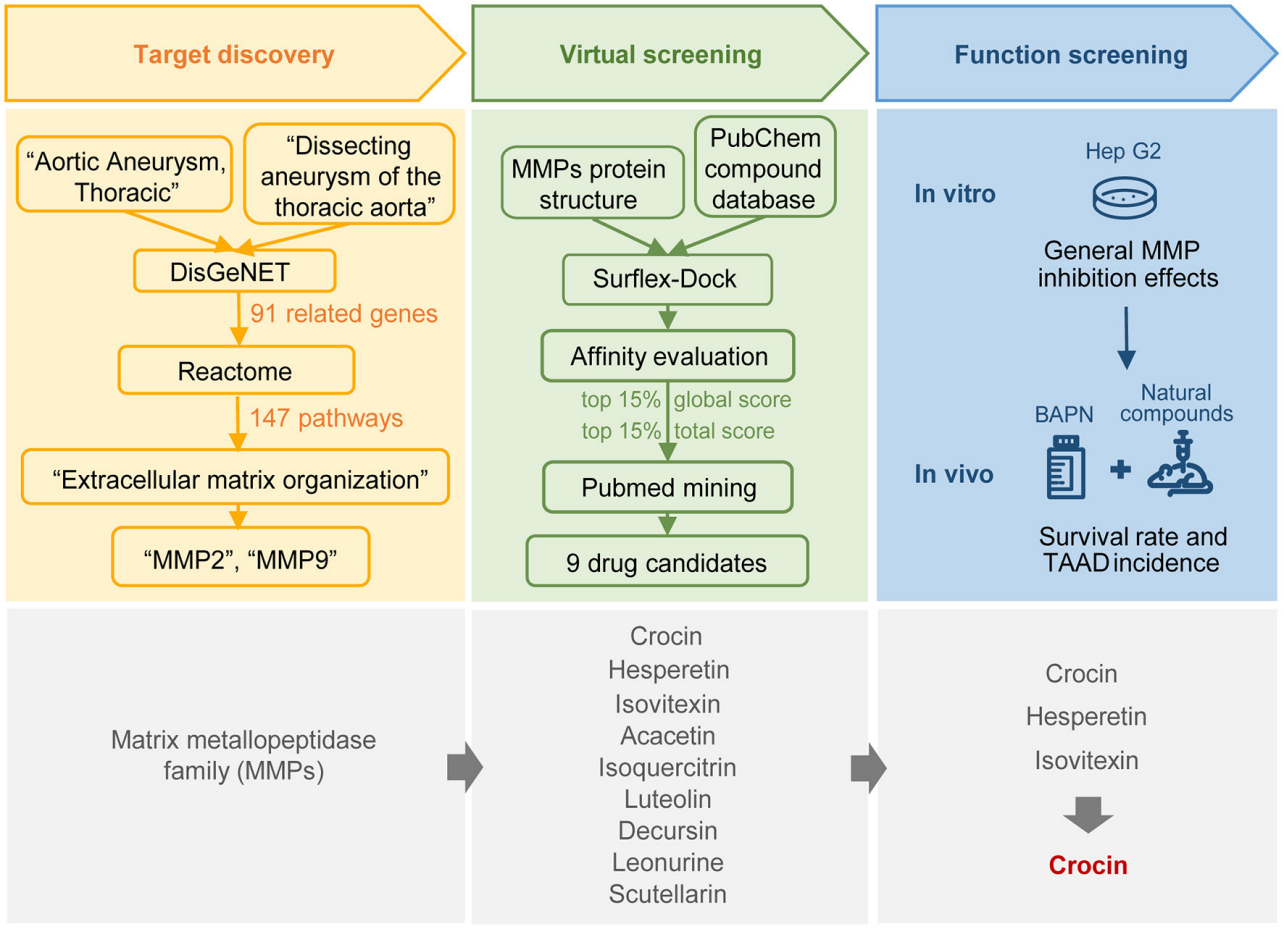
Flow diagram of TAAD potential drugs screening.

**Figure 2:**
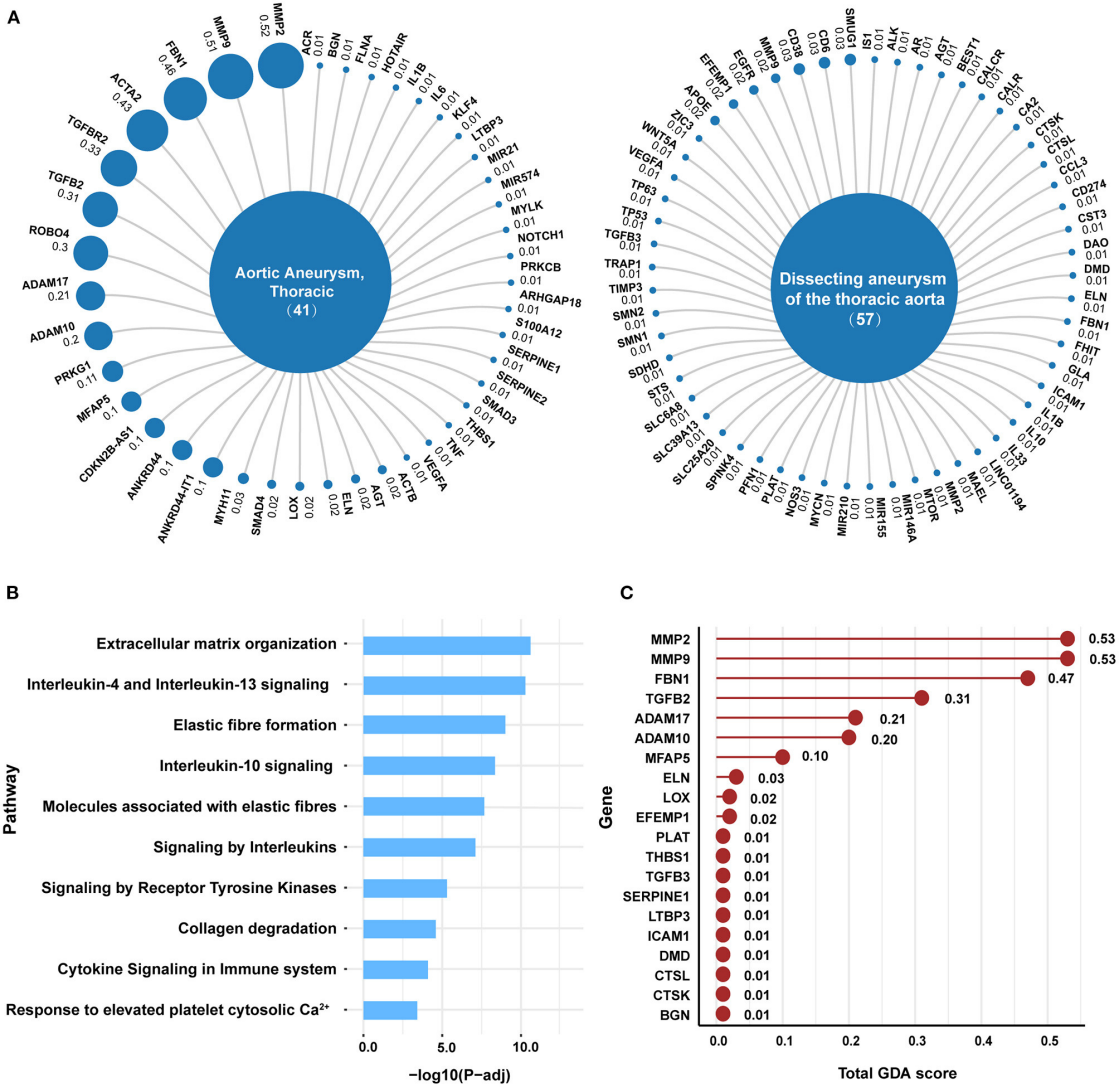
Unbiased bioinformatic analysis discerns MMPs as the key targets of TAAD. **(A)** Disease–target network of TAAD-related genes collected from DisGeNET. Each blue circle represents a gene, and the GDA score from DisGeNET is correlated with the size of the circle. Different distance between disease and genes conveys no meaning. **(B)** Top ten pathways are ranked by corrected *P*-values related to above genes in Reactome. **(C)** 20 genes of “Extracellular matrix organization” pathway are sorted by total GDA score.

Figure 1 shows the flow diagram of the selection process. There are two entries, “Aortic Aneurysm, Thoracic, C0162872” and “Dissecting aneurysm of the thoracic aorta, C0729233” for TAAD in the DisGeNET database, representing a total of 91 non-redundant TAAD-associated genes (41 for C0162872 and 57 for C0729233; Figure 2A, Supplementary Tables 1, 2). The results of the gene-set analysis showed that there were 147 pathways with a *P*-value < 0.05 in Reactome (Supplementary Table 3, Supplementary File 1). The top pathway is “Extracellular matrix organization” (Reactome id: R-HSA-1474244) with *P*-value = 4.91e-14 and *FDR* = 3.20e-11 (Figure 2B). There are 20 TAAD-related genes related to extracellular matrix (ECM) organization. We further checked the rank of these 20 genes in DisGeNET and found that matrix metalloproteinase family genes *MMP2* (total GDA score = 0.53) and *MMP9* (total GDA score = 0.53) had the two highest rankings (Figure 2C, Supplementary Table 4). Considering the role of the MMP family in ECM and TAAD (10), we focused on the MMP family as candidate targets for subsequent drug screening.

### Potential Natural MMP Inhibitors Were Identified by Artificial Intelligence Virtual Screening

**Figure 3:**
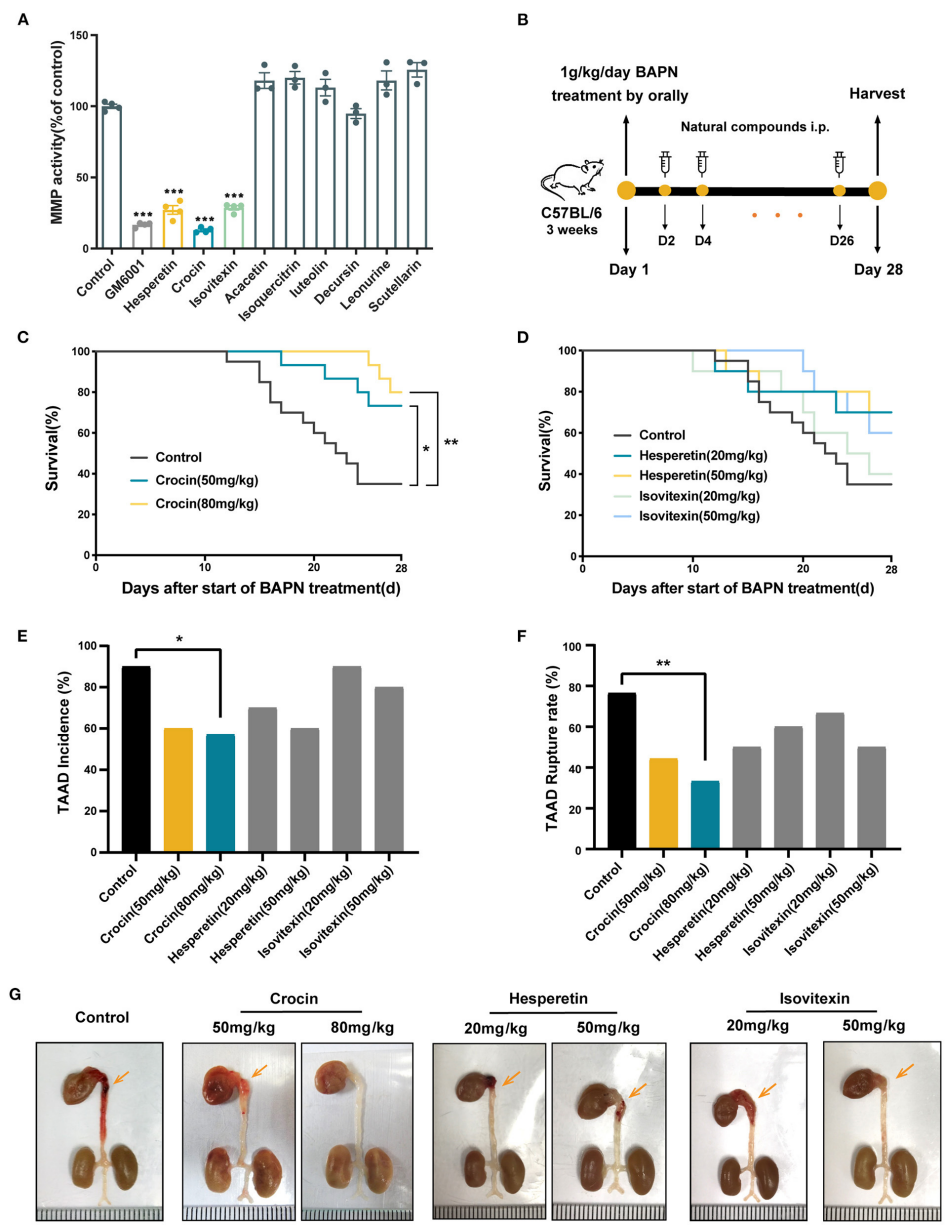
Crocin inhibits general MMP activity and prevents the occurrence and rupture of TAAD in early administration model. **(A)** Functional screening of MMPs activity inhibition of 9 natural substances in Hep G2. Hep G2 cells were incubated for 36 h after treated with 50 μM compounds, and the general MMP activity in cell supernatant was measured by general MMP activity kit. Values are expressed as fold change with respect to untreated control. *n* = 3–4, values were expressed as the mean ± SEM, and analyzed by unpaired *T*-test. ***p < 0.001 vs. control group. **(B)** Experimental process of TAAD model establishment and preventional drug delivery. **(C,D)** Survival curves of mice in each group. Control, *n* = 20; crocin 50 mg·kg^−1^, *n* = 15; crocin 80 mg·kg^−1^, *n* = 14; hesperetin 20 mg·kg^−1^, *n* = 10; hesperetin 50 mg·kg^−1^, *n* = 10; isovetixin 20 mg·kg^−1^, *n* = 10; isovetixin 50 mg·kg^−1^, *n* = 10. Values were analyzed by Log-rank (Mantel-Cox) test (conservative), *p < 0.05, **p < 0.01 vs. control BAPN group. **(E)** Bar graph showed the incidence of TAAD formation of either vehicle or natural compounds treated mice. Values were analyzed by Fisher's exact test, *p < 0.05 vs. control BAPN group. **(F)** Bar graph showed the incidence of rupture in all aortas with TAAD of either vehicle or natural compounds treated mice. Values were analyzed by Fisher's exact test, *p < 0.05, **p < 0.01 vs. control BAPN group. **(G)** Representative images of the whole aortas of either vehicle or natural compounds treated mice for 28 days. Orange arrows represent aortic dissection or aneurysm.

We first extracted all the available structures for the MMP family from the Protein Data Bank (PDB) database. The active site ligand of MMP (1–14) was used as prototype molecules to construct the 3D structure of the protein receptor. The Surflex-Dock module in Sybyl-x2.0 software was then used to map small molecule compounds from the PubChem compound database to the prototype molecules. Finally, we evaluated and ranked the results using scoring function and obtained nine natural compounds with global score and total score values in the top 15%. We next determined the effect of these nine compounds on MMP activity *in vitro*. We first evaluated general MMP activity in three common MMP hyperexpression cell lines: HeLa, Hep G2, and MDA-MB-231. Hep G2 demonstrated high MMP activity and was the most sensitive to GM6001, a broad spectrum MMP inhibitor, thus we selected Hep G2 cells for subsequent experiments (Supplementary Figure 1). Hep G2 cells were then treated with the nine natural compounds at a concentration of 50 μM; GM60001 was used as positive control. Crocin (a carotenoid pigment of saffron), isovitexin (a flavonoid isolated from rice hulls of *Oryza sativa*), and hesperetin (a predominant flavonoid in lemons and oranges) displayed significant inhibition of general MMP activity (Figure 3A). Thus, we selected these three molecules for further screening.

### *In vivo* Functional Screening Identified Crocin as a Specific Anti-TAAD Molecule

To further confirm the anti-TAAD effect of above compounds *in vivo*, we constructed a β-Aminopropionitrile (BAPN)-induced TAAD mouse model. Using an early administration regimen, 3-week-old C57BL/6 mice were given 1 g·kg^−1^ BAPN per day in water for 28 days, and the three natural compounds or solvent alone (control) was administered intraperitoneally every other day from the first day to the end point (Figure 3B). Two dose groups were established for each compound. The doses were determined from previous studies (11–19). At the end of the 28th day, the survival rate in 50 mg·kg^−1^ crocin group and 80 mg·kg^−1^ crocin group was significantly higher compared to the control, while there was no significant difference between hesperidin and isovitexin groups with the control group (Figures 3C,D). The 80 mg·kg^−1^ crocin group also prevented the incidence of TAAD (Figure 3E). The TAAD rupture rate was significantly lower in the 80 mg·kg^−1^ crocin group than in the control (Figure 3F). In addition, general observation of all surviving mice showed that the extent and scope of TAAD in animals that received crocin treatment was better than those in the control group (Figure 3G). The above results suggest that the early administration of crocin could prevent the occurrence and development of TAAD in mice.

### Crocin Improved Extracellular Matrix Metabolic Disorder of Aorta in an Early Administration Model

**Figure 4:**
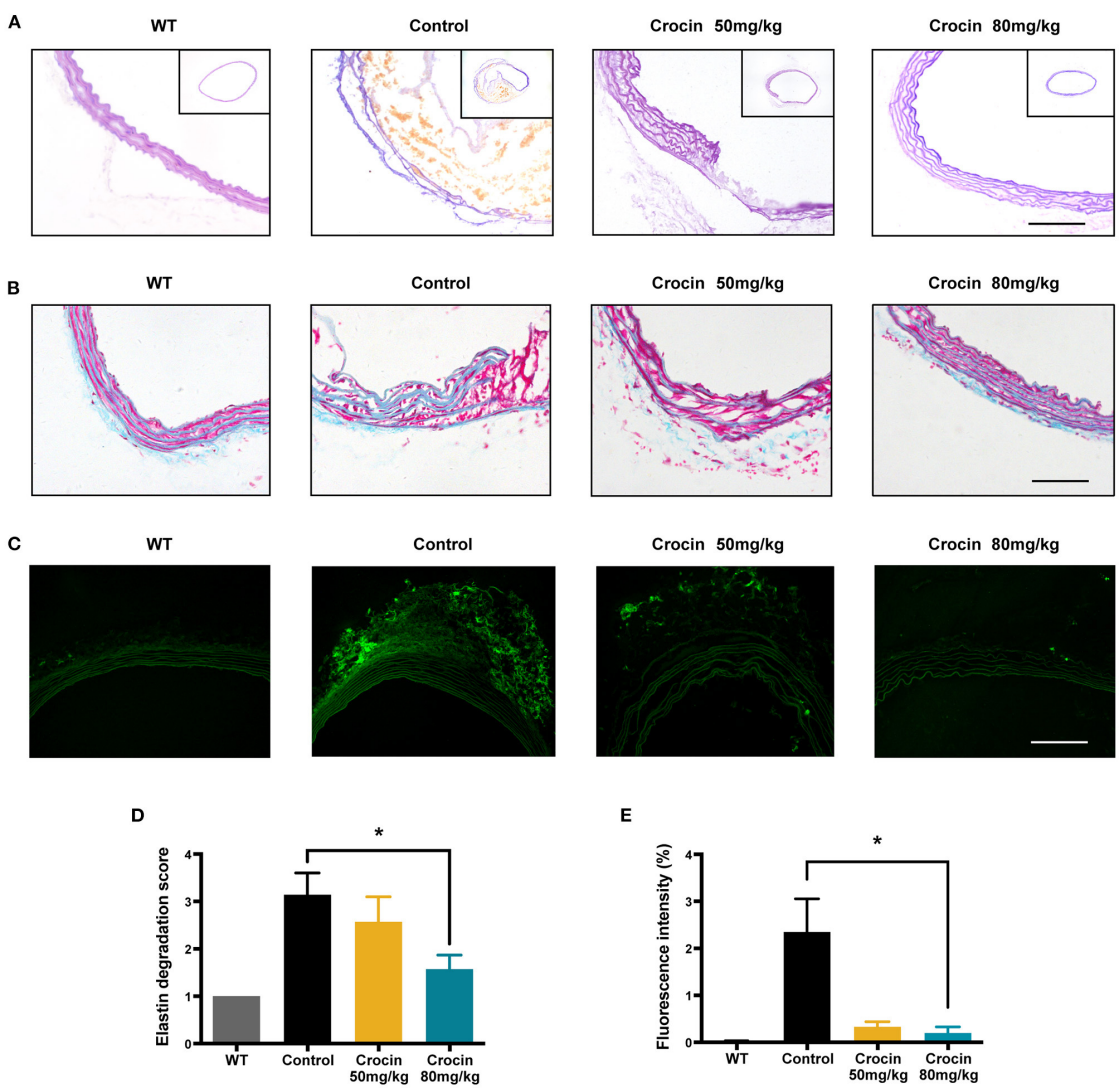
Early intervention of crocin improved extracellular matrix metabolic disorder in TAAD mice. **(A)** Representative images of elastin staining of thoracic aortas from WT group (health seven-week-old C57BL/6 mice), control group and crocin-treated groups for 28 days; scale bar: 50 μm. **(B)** Representative images of Gomoris staining of thoracic aortas from WT group (health seven-week-old C57BL/6 mice), control group and crocin-treated groups for 28 days; scale bar: 50 μm. **(C)** *in situ* zymography of aortas from WT group (health seven-week-old C57BL/6 mice), control group and crocin-treated groups for 28 days; scale bar: 50 μm. **(D)** Elastin degradation score in WT, control and crocin-treated groups, *n* = 7. **(E)** Fluorescence intensity in WT, control and crocin-treated groups, *n* = 3.Values were expressed as the mean ± SEM, and analyzed by unpaired *T*-test. *p < 0.05 vs. control group.

Representative elastin staining, Gomori's staining and *in situ* fluorescence staining images of the thoracic aorta following BAPN induction and crocin administration were shown in Figures 4A–C. Compared to control group, elastic fiber staining in 80 mg·kg^−1^ crocin group displayed significant improvement in intact vessel morphology, as well as less elastic fiber breakage (Figure 4A). Gomori's staining indicated that 80 mg·kg^−1^ crocin preserved more smooth muscle in the aorta (Figure 4B). As showed in Figure 4D, the elastin degradation score was significantly decreased in 80 mg·kg^−1^ crocin group. Results of the artificial intelligence (AI) molecular docking experiment revealed that crocin could bind with MMP1, MMP2, MMP3, MMP10, and MMP14 (Supplementary Table 5). MMP2, also known as gelatinase A, has been shown to play a crucial role in TAAD (Figure 2C). To gain insights into the MMP2 activity on aorta, gelatin activity was investigated by *in situ* fluorescence staining using DQ-*gelatin*. Mean fluorescence intensity revealed that gelatin activity was significantly inhibited on the aortic wall in 80 mg·kg^−1^ crocin group (Figure 4E). Crocin blunted elastin degradation and down-regulated MMP expression after BAPN administration.

### Crocin Exhibited Therapeutic Effect on TAAD in BAPN-Induced Model

**Figure 5:**
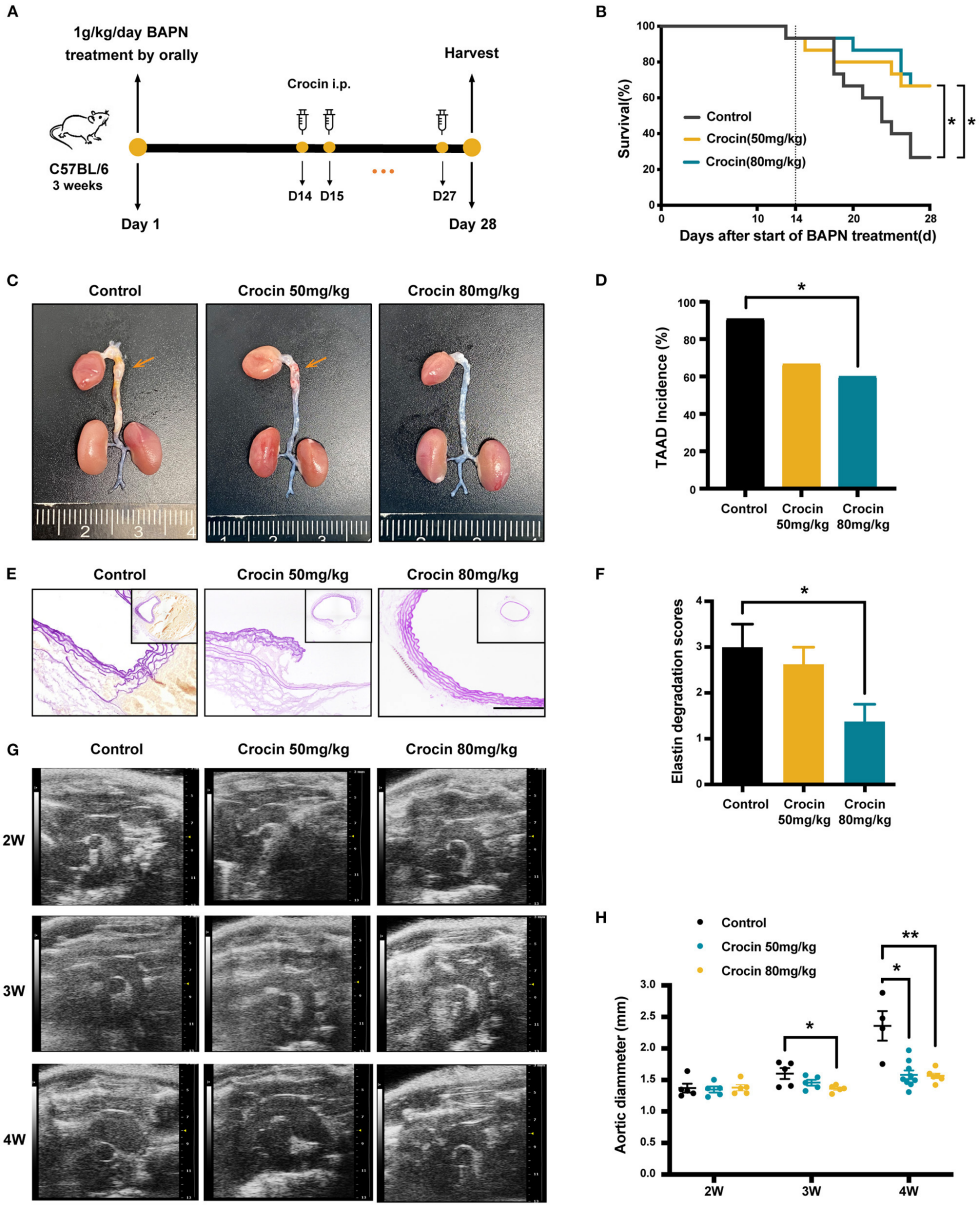
Crocin arrests the progression and rupture of damaged aorta into TAAD. **(A)** Experimental process of TAAD model establishment and therapeutic drug delivery. **(B)** Survival curves of mice in each group. Control, *n* = 15; crocin 50 mg·kg^−1^, *n* = 15; crocin 80 mg·kg^−1^, *n* = 15; Values were analyzed by Log-rank (Mantel-Cox) test (conservative),*p < 0.05 vs. control BAPN group. **(C)** Representative images of the whole aortas for 28 days. The orange arrows represent aortic dissection or aneurysm. **(D)** Bar graph showed the incidence of TAAD formation of either vehicle or crocin-treated mice. Values were analyzed by Fisher's exact test, *p < 0.05 vs. control BAPN group. **(E)** Representative images of elastin staining of thoracic aortas on day 28; scale bar: 50 μm. **(F)** Elastin degradation score in WT, control and crocin-treated groups, *n* = 7. **(G)** Representative ultrasound images and aortic diameter **(H)** of aortas for 2, 3, and 4 weeks; *n* = 4–9, *p < 0.05, **p < 0.01 vs. control BAPN group. Values were expressed as the mean ± SEM, and analyzed by unpaired *T*-test. *p < 0.05 vs. control group.

To confirm the therapeutic effects of crocin, we adjusted the timing of dosing in the mouse model. Our previous study found that 4 weeks BAPN administration caused 87% of the animals to develop TAAD (20). Moreover, endothelial cell damage, elastic fiber disruption, and a 15% TAAD rupture rate was already present by the end of the second week (21). These results suggested that pathological changes were already prevalent in mice treated with 1 g·kg^−1^·day^−1^ BAPN on the 14th day. Therefore, we chose day 14 as an appropriate intervention time point. We designed two groups of crocin: 50 mg·kg^−1^ and 80 mg·kg^−1^. Mice were given 1 g·kg^−1^ BAPN per day in water for 28 days, and crocin or solvent alone (control) was administered intraperitoneally everyday from day 14 to the end point (Figure 5A). Survival rates of both two crocin groups were significantly higher than the control group (Figure 5B). Gross examination of the aorta displayed less dilation and less TAAD formation after crocin treatment (Figures 5C,D). Elastic fiber staining exhibited significant dissection in the control group, with thrombus filling the false lumen. In the crocin-treated groups, the morphology of the aortic vessel wall was improved (Figure 5E). As showed in Figure 5F, the elastin degradation score was significantly decreased in 80 mg·kg^−1^ crocin group. In addition, ultrasound results performed during weeks 2–4 showed that crocin significantly inhibited dilation of the aorta (Figures 5G,H). These results suggest that crocin can not only prevent the development of TAAD at an early stage but also have a therapeutic effect on TAAD pathological changes.

### Crocin May Inhibit MMP2 Activity by Direct Binding

**Figure 6:**
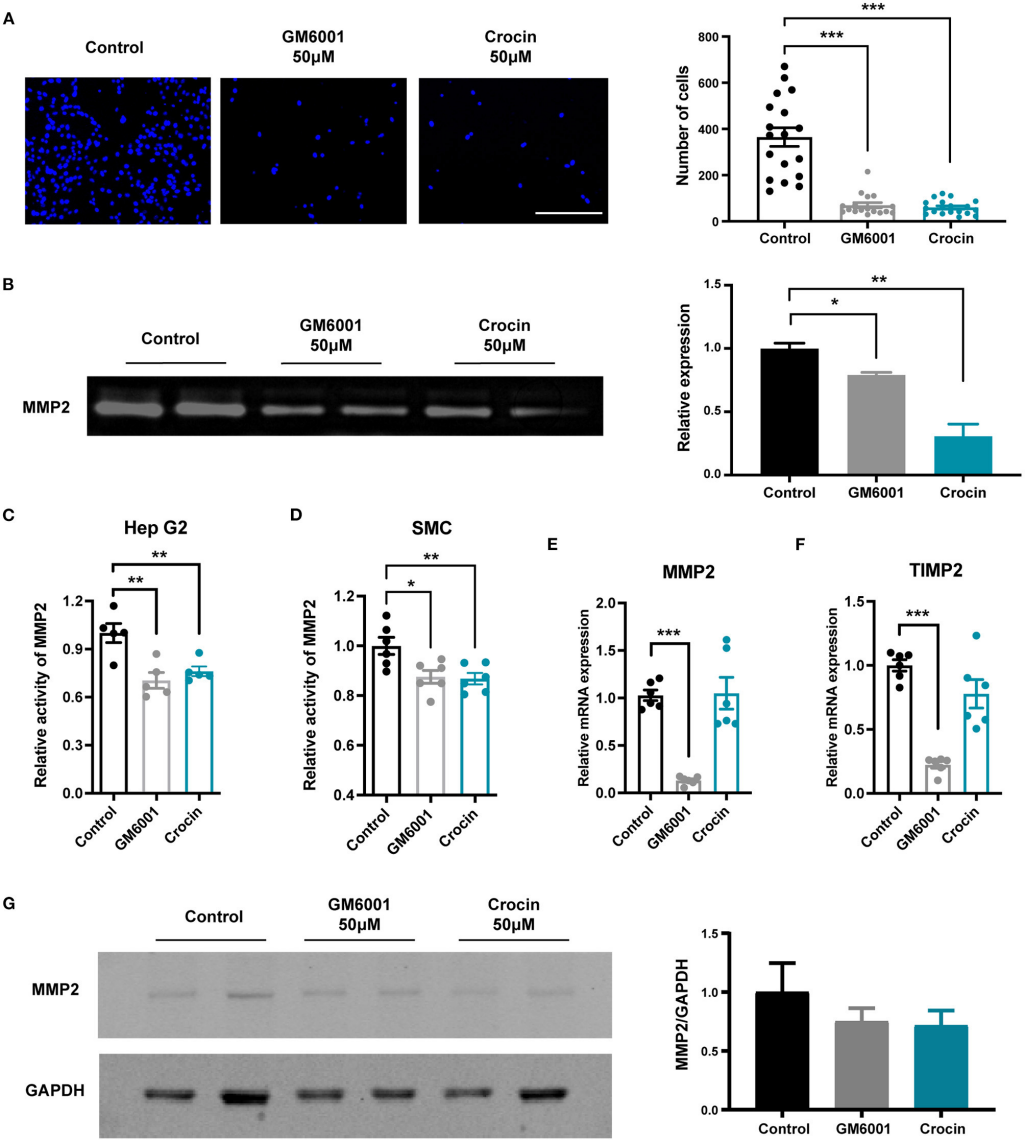
Crocin inhibits the activity of MMP2 but does not affect its expression. **(A)** Representative pictures and cell count for migration of SL4 cell detected by transwell migration assay. Scale bar: 200 μm; *n* = 17–19. **(B)** MMP2 activity measured by gelatin zymography (normalized to GAPDH), *n* = 3. **(C)** MMP2 relative activity by MMP2 Assay kit. Hep G2 were incubated for 36 hafter treated with 50 μM crocin or GM6001, then cell supernatant was collected for detection, *n* = 5. **(D)** MMP2 relative activity by MMP2 Assay kit. Mouse primary aortic smooth muscle cells were incubated for 36 hafter treated with 50 μM crocin or GM6001, then cell supernatant was collected for detection, *n* = 6. **(E,F)** Real-time PCR analysis of MMP2 and TIMP2 mRNA levels of Hep G2 after 36 h 50 μM crocin treated, *n* = 6. **(G)** Western blot analysis of MMP2 expression in Hep G2 cells, which were treated with PBS or 50 μM crocin for 36 h (normalized to GAPDH). Values were expressed as the mean ± SEM, and analyzed by unpaired *T*-test. *p < 0.05 vs. control group. **p < 0.01 vs. control group, ***p < 0.001 vs. control group.

To fully demonstrate the inhibitory effect of crocin on MMP and to verify the effect in different cell lines, we selected another highly invasive human intestinal cancer cell line SL4 for migration invasion assays. The number of cells crossing the matrigel was significantly reduced in crocin-treated groups, demonstrating that crocin could reduce extracellular matrix degradation by inhibiting MMP activity (Figure 6A). To further clarify the MMP2 activity inhibition of crocin, we collected culture supernatants from Hep G2 cells for gelatin zymography and MMP2 activity measurements. The results of gelatin zymography (Figure 6B) and MMP2 activity (Figure 6C) demonstrated that crocin significantly inhibited MMP2 activity secreted by Hep G2 cells. MMP2 is predominantly secreted in the vascular wall by vascular smooth muscle cells and endothelial cells, of which vascular smooth muscle cells are the main source (22). We detected the MMP2 activity on mice primary aortic smooth muscle cells by using MMP2 activity kit. The result indicated that crocin could significantly inhibit the MMP2 activity secreted by smooth muscle cells as well (Figure 6D). This is consistent with what has been found in Hep G2 (Figure 6C).

**Figure 7:**
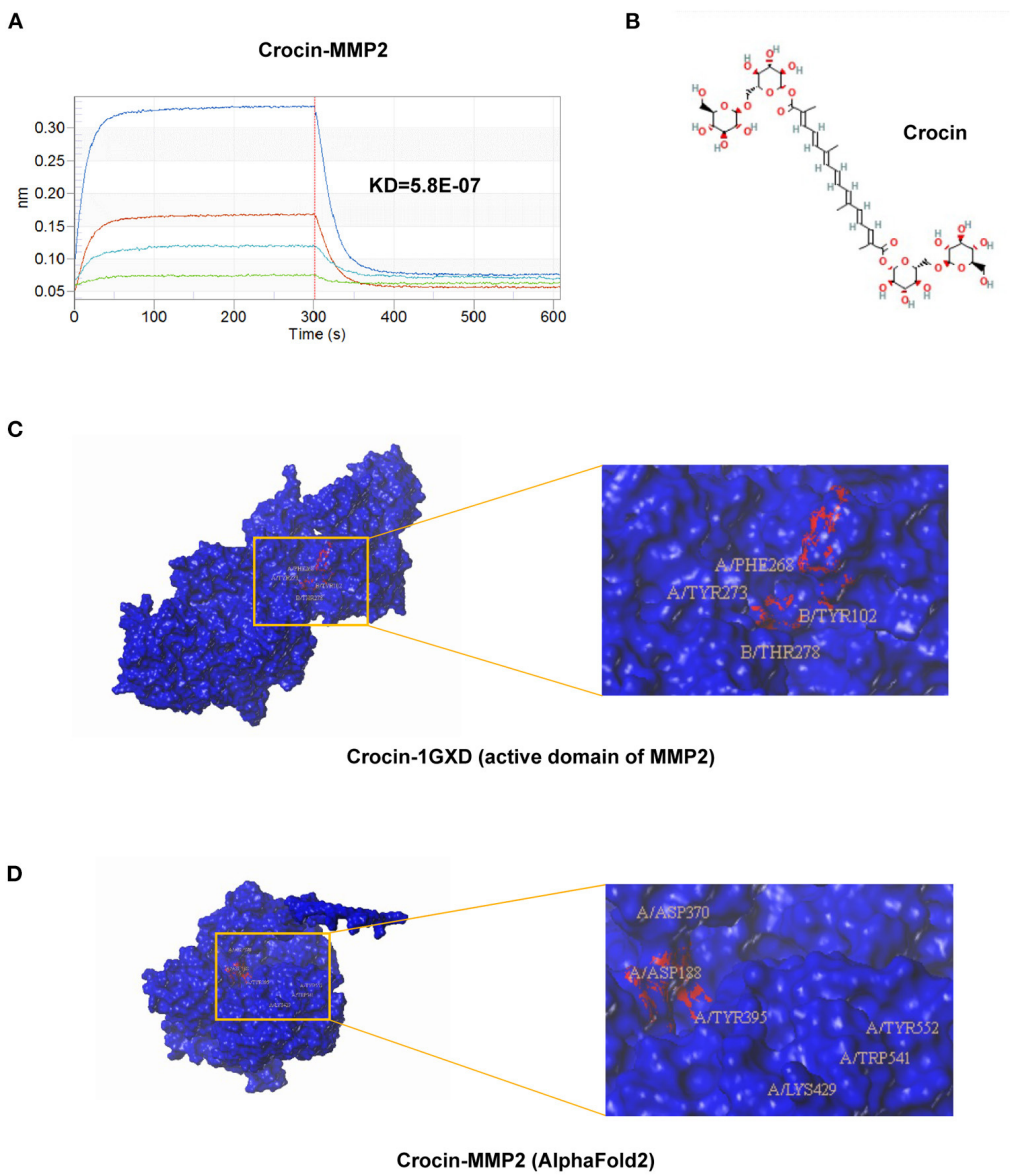
Possible binding mode of Crocin to MMP2. **(A)** BLI detection for the affinity of crocin with MMP2. **(B)** 2D structure of crocin was obtained from PubChem (https://pubchem.ncbi.nlm.nih.gov/compound/5281233). **(C)** Schematic of the predicted binding site of crocin with MMP2 active domain 1GXD obtained from PDB database by Sybyl-x2.0 software (http://www.rcsb.org/). **(D)** Schematic of the predicted binding site of crocin with MMP2 by Sybyl-x2.0 software. Complete AlphaFold2 three-dimensional (3D) structure of MMP2 was downloaded from DeepMind algorithm AlphaFold2 system (https://deepmind.com/).

We confirmed the role of crocin on MMP2 activity; however, whether the inhibitory effect was partially due to the regulation of MMP2 or tissue inhibitors of matrix metalloproteinases (TIMPs) expression was unknown. To investigate the specific mechanisms by which crocin inhibits MMP2 activity, we extracted mRNA and protein from cells treated with 50 μM crocin for 36 h. Gene expression of *TIMP2* and *MMP2* was detected by real-time PCR, and protein expression of MMP2 was detected by western blotting. These results indicated no significant difference between the experimental and control groups (Figures 6E–G). Combined with the previously predicted binding modes and sites of the natural compounds and MMP2, evidence suggested that crocin may directly bind to MMP2 and inhibit its activity. Hence, we determined the binding affinity between crocin and MMP2 using the biofilm interference technique (BLI), which revealed that crocin had a very high affinity for MMP2 (Figure 7A). To further study the specific binding sites of crocin and MMP2, we downloaded the structure diagram of crocin from the PubChem Compound Database (Figure 7B) and established a predicted binding mode map based on the previous Surflex-Dock results in which the active domain of MMP2 1GXD from the PDB database was used (Figure 7C). The other complete 3D structure of MMP2 (with full length 660 AA) was predicted by AlphaFold2, a powerful and accurate AI algorithm engine for protein 3D structure prediction (23). The AlphaFold network utilized a multiple sequence alignment (MSA) to represent 3D information of residue pairs and generate 3D structure coordinates based on the representation. Then Surflex-Dock was employed to identify active sites of the complete protein structure and ligand-protein (Crocin-MMP2) interaction (Figure 7D). The residues interact with crocin were further analyzed.

Based on the above results, we considered that crocin ameliorated the development of TAAD primarily by targeting the active site of MMP2 directly to inactivate it, rather than affecting the expression of MMP2 or TIMPs.

## Discussion

In this study, we first determined that the MMP family is the most important protein group in the pathogenesis of TAAD and used it as the target for natural compound screening, from which we identified crocin. We verified the therapeutic ability of crocin in a BAPN-induced TAAD mouse model *in vivo* and its inhibitory effect and binding mode on MMP activity *in vitro*. The results suggest that a possible therapeutic mechanism in which crocin directly binds to MMP2 to inactivate it, preventing the degradation of the extracellular matrix, disruption of elastic fibers, and further expansion and rupture of TAAD. This is consistent with our initial prediction generated by an artificial intelligence approach, illustrating the possibility and reliability of using AI to assist in new drug development.

Changes in the structural components of ECM affect the molecular signaling pathways that regulate the homeostasis of the aortic wall and aortic function (7). MMPs are the most important proteases involved in ECM degradation (24). MMP is a family of endopeptidases that play biological roles dependent on zinc and calcium ions. In TAAD, high MMP expression and activity may destroyed the stability of the arterial wall, leading to the occurrence of thoracic aortic aneurysm/dissection and making it prone to rupture (25). It has been reported that when aortic dissection occurs, the serum levels of MMP1, MMP2, MMP3, MMP8, and MMP13 are increased, and the expression of MMP9 in the cytoplasm of smooth muscle cells in the aortic wall is enhanced (26–28). Overexpression of MMP2 in smooth muscle cells and MMP9 in neutrophils and macrophages can cause degenerative changes in the middle layer of the artery in aortic aneurysms (29–31), and knockout of MMP2 and MMP9 can inhibit the development of thoracic aortic aneurysms (30). A previous study by our team also found that IL-3 stimulated macrophages to produce MMP12 through JNK- and ERK1/2-dependent AP-1 pathways, thus promoting TAAD formation (32). These studies demonstrate that multiple members of the MMP family are involved in the formation of TAAD, which also supports the credibility of focusing on MMPs as a therapeutic target for TAAD through unbiased bioinformatics analysis in this study.

The most promising MMP inhibitor for the treatment of aneurysms is doxycycline, a semi-synthetic compound of tetracycline, which has been shown to effectively inhibit aortic dilation in animal models of abdominal aortic aneurysm (33). However, it was shown to be ineffective at reducing the growth of small abdominal aortic aneurysms in patients in a 2-year clinical trial last year (34). Furthermore, administration of doxycycline has been shown to lead to dose-dependent systemic side effects, such as gastrointestinal tract disturbances, dental discoloration, and cutaneous photosensitivity in some patients. On the other hand, natural products are an important source for drug development because of their structural diversity, high multi-target activity, and low toxic side effects (35). Resveratrol (a polyphenol from the skin of red grapes) has previously been reported to significantly inhibit aortic root dilation in MFS mice by increasing NF-κB activity to reduce miR-29b, even significantly better than losartan (a commonly used Angiotensin II receptor antagonists' class of antihypertensive drugs in TAAD) (36). It has also been reported that green tea phenol and tanshinone, a natural extract of *Salvia miltiorrhiza*, can effectively inhibit MMP-2 and MMP-9 activity to reduce the development of abdominal aortic aneurysms and that tanshinone has a better safety profile than doxycycline (37). Therefore, this suggests that we could find safe and effective anti-TAAD drugs in natural products.

Crocin is the main pigment component of saffron *(Crocus sativus L.)* used in traditional Chinese medicine, with a medical history of thousands of years. In recent years, increasing attention has been paid to the anti-inflammatory, antioxidant, anti-tumor, and other active effects of crocin (38). In the field of cardiovascular diseases, studies have reported that crocin can inhibit the increase of intracellular Ca^2+^ in smooth muscle cells (39–41) and lower blood pressure in normotensive and desoxycorticosterone acetate-induced hypertensive rats. In addition, crocin can have an anti-atherosclerotic effect by reducing apoptosis in macrophages induced by Ox-LDL and inhibiting the formation of foam cells (42). A few articles have reported that crocin can inhibit the activity of MMP1 and MMP2, but the research was limited to cell lines and did not explore the specific binding mode between crocin and MMP (43). However, in this study, we demonstrated that crocin can inhibit MMP activity both *in vitro* and *in vivo*, and proposed the possibility of crocin binding to MMP2 directly.

This study has some limitations. To avoid the first-pass effect caused by oral administration and improve bioavailability, we adopted intraperitoneal injection as the administration method instead of an oral one. In future studies, oral administration should be considered. Moreover, combined with previous reports and the multi-target properties of natural compounds, crocin may have other mechanisms for the treatment of TAAD, which would require further study.

In summary, this study found that crocin, a natural compound, can prevent the occurrence and development of TAAD by inhibiting the activity of MMPs, providing new approaches and therapeutic targets for the clinical prevention and treatment of TAAD.

## Materials and Methods

### Bioinformatic Analysis

To investigate candidate drug targets of TAAD, we first performed a bioinformatics analysis. The main idea was to search for the biological pathways in which TAAD-related genes are involved and select candidate genes from these pathways. Initially, we searched DisGeNET, an integrative database that collects genes and variants associated with human diseases, for TAAD-related genes (44). We then employed a gene-set analysis (hypergeometric test) implemented in KOBAS 3.0 (https://pubmed.ncbi.nlm.nih.gov/34086934/) to search Reactome (45). We utilized a *P*-value < 0.05 as a significance cutoff and ranked hits on it. We ranked TAAD-related genes from the top pathways by summing their Gene-Disease Association (GDA) score from DisGeNET entries. Genes with the top ranks were considered candidate key targets.

### Surflex-Dock Virtual Screening

The protein structures of MMP (1–14) were obtained from the RSCB PDB database (46). The docking pocket was generated after a series of steps (including extraction ligand binding, dehydration, addition of hydrogen and generating the protomol) and saved in SFXC format using Sybyl-x2.0 software (47, 48). The SDF format of small molecule structures was obtained from the Pubchem Compound Database (https://pubchem.ncbi.nlm.nih.gov/), and then energy minimization and optimization were conducted using Sybyl-x2.0 software. The sybyl_mol2 format was saved for subsequent molecular docking (47). Surflex combines the scoring capabilities of the Hammerhead docking system with a search engine that relies on surface-based molecular similarity approaches to rapidly generate putative poses suitable for molecular fragments, and the docking results are considered reliable and accurate (49).

The Surflex-Dock (SFXC) docking mode was used, and the procedure was conducted as previously described (50). The interconnection parameters were set to default values. The global score of each molecule was obtained using Hammerhead's empirical scoring function, and molecules with scores in the highest 15% were used as candidates for the next calculation to obtain a total score. Surflex-Dock scores (total scores) represent binding affinities (51). The total score values comprehensively considered the polarity effect, hydrophobic effect, and other factors, the higher the value, the stronger the binding force between a ligand and receptor, and higher the activity. We then carried out the next step, biological function verification, using the top 15% hits based on total scores.

### Cell Culture and Treatment

Mouse primary aortic smooth muscle cells were obtained from aorta of eight-week-old C57BL/6 mice. Hep G2 cell line and SL4 cell line were purchased from American Type Culture Collection (ATCC, USA).These cells were grown at 37°C with 5% CO_2_ in DMEM (Gibco, USA) with 10% FBS (Gibco, USA) and 1% penicillin and streptomycin (Gibco, USA). Hep G2 cells and mouse primary aortic smooth muscle cells were seeded in six-well plates at a density of 1 × 10^6^ per well, grown to a suitable density, starved with serum-free medium for 12 h, and then treated with 50 μm crocin (Chengdu Biopurify Phytochemicals Ltd, China) or 50 μm Gm6001 (MedChemExpress, USA). Crocin was solubilized in DMSO and diluted further into working concentrations before experiments by PBS. No treatment was used as the negative control. After 36 h, cell supernatants were collected and centrifuged for 5 min at 1,500 rpm for gelatin zymography or MMP activity detection. Cell layers were rinsed with PBS three times, harvested with 0.25% trypsin, and centrifuged for 5 min at 1,500 rpm for real-time PCR and western blotting, as described below.

### Enzyme Activity Assays

Generic MMP activity was measured using the SensoLyte^®^ 520 Generic MMP Activity Kit Fluorimetric (Anaspec, CA). Cell supernatants described above were centrifuged at 1000 g for 15 min at 4°C, and the precipitate was discarded. In a 96-well plate, 50 μL/well of the MMP-containing sample was added. A substrate control (50 μL/well) and positive control (50 μL/well) were also set up. The generic MMP substrate (Component A) was diluted 1:100 in assay buffer (Component D), and 50 μL/well was added to each sample and control well. The reagents were mixed by shaking the plate gently for 30 s. The reaction was incubated at 15–25°C for 30–60 min in the dark; 50 μL/well of stop solution (Component E) was then added and mixed, and the fluorescence intensity was measured at Ex/Em = 490 nm/520 nm.

MMP2 activity was determined using the SensoLyte^®^ Plus 520 MMP-2 Assay Kit (Anaspec, CA). Supernatants were collected and centrifuged as before. The MMP2 standard (10 mg/mL, Component B) was diluted 50-fold in assay buffer (Component C) to obtain a concentration of 200 ng/mL. We then made six 2-fold serial dilutions using assay buffer. Next, 100 μL/well of MMP2 standards, samples, and blank controls were added to the microplate pre-coated with monoclonal anti-human MMP2 antibody (Component A). The plate was then covered with an adhesive cover strip (Component H) to prevent evaporation. The plate was incubated on a plate shaker (40-100 rpm) at RT for 2 h. The 10X wash buffer (Component D) was then diluted to 1X in deionized water. The wells were then washed four times with 200 μL of 1X wash buffer. The MMP2 substrate (Component F) was diluted 200-fold in assay buffer (Component C), and the reaction incubated at RT in the dark for 1 h to 24 h. The fluorescence intensity was then measured at Ex/Em = 490 nm/520 nm.

### Mice and TAAD Model

All protocols were approved by the Institutional Animal Care and Use Committee of Capital Medical University. The mice model of TAAD was constructed as described previously (20, 21). Gender difference was presented in AAD mice model induced by beta-aminopropionitrile (BAPN) (52, 53). To exclude the influence of gender and obtain higher incidence of TAAD, we selected male mice as the experimental subject.3-week-old male C57BL/6 mice were purchased from Beijing HFK Bioscience Co., Ltd. All mice were housed in an SPF-level animal laboratory at Beijing Anzhen Hospital. Mice were fed a regular diet and freshly prepared β-aminopropionitrile (BAPN) (Sigma Aldrich, USA) dissolved in drinking water (1 g ·kg^−1^·d^−1^) was administered for 28 days. During this experiment, all deaths were recorded, and survival curves were plotted (no unexpected deaths occurred, as all deaths were attributed to rupture of the aortic aneurysm or dissection). At the end of the experiment, the surviving mice were euthanized by bleeding out under deep anesthesia, the entire aorta was dissected to observe the gross condition and photographed, and the excised aorta was immersed in 4% paraformaldehyde for 24 h and 30% sucrose solution over 48 h to dehydrate and mount. The aortic arch was cut off, embedded in OCT, frozen at −80°C, and then was cut into 7-μm-thick frozen sections by Leica CM1905.

### Elastin Staining

Elastin in control and TAAD mouse aortas was stained with an Elastic-Fiber Stain Kit utilizing Gomori's aldehyde-fuchsin staining procedure (MXB Biotechnologies, China). Frozen aortic sections were dried at room temperature for 15 min before being rinsed three times in PBS. They were incubated in Lugol's iodine solution for 5 min, then washed twice in PBS before being treated with sodium thiosulfate for 5 min. Sections were rinsed for 5 min with running tap water, then soaked in 70% ethanol for 10 min, then incubated with aldehyde-fuchsin for 10 min, washed with 70% ethanol until no longer bleached, then stained with acid orange G for 10s. Finally, neutral gum was used to seal the slices, which were then examined under a microscope. As described previously (54, 55), elastin degradation was classified based on the elastin score: grade 1, intact, well-organized elastic layer; grade 2, elastic layer with some interruptions and fractures; grade 3, severe elastin fragmentation or loss; and grade 4, severe elastin degradation with visible false lumen or rupture sites.

### Collagen Fibers Staining

Collagen and muscle fibers in control and TAAD mouse aortas were stained with a Masson Trichrome Stain Kit (Leagene, USA). Frozen aortic sections were dried at 15–25°C for 15 min and rinsed in PBS three times. Equal volumes of hematoxylin and Weigert's Iron (Parts A and B) were mixed. Sections were rinsed in the prepared Weigert's Iron-hematoxylin for 5–10 min, differentiated with acidic ethanol for 2 s, and washed with water. Sections were rinsed in Masson blue liquid to make nuclei turn to blue and washed with distilled water for 1 min before staining with Ponceau red fuchsin solution for 5–10 min. During the above operation, a weak acid working solution at a ratio of distilled water:weak acid solution = 2:1 was prepared, and sections were rinsed in it for 1 min, followed by washing with phosphomolybdic acid solution for 1–2 min. Sections were washed with the prepared weak acid working solution for 1 min, dyed directly into the aniline blue staining solution for 1–2 min, washed with the prepared weak acid working solution for 1 min, and quickly dehydrated in 95% ethanol. Sections were dehydrated with anhydrous ethanol three times for 5–10 s each time and rendered transparent by xylene three times for 1–2 min each time before being sealed with neutral gum.

### Situ MMP Zymography

Situ MMP zymography was measured by Gelatinase/Collagenase Assay Kit (Invitrogen, Carlsbad, CA), as previously described (56). Freshly cut frozen aortic sections were dried for 15 min at 15–25 degrees Celsius, rinsed three times in PBS to remove OCT, and incubated for 2 h in 20 g/ml DQ-labeled gelatin in 50 mM Tris-HCl, 50 mM NaCl, 10 mM CaCl2, pH 6.8, at 37°C in the dark. In the presence of 5 mM EDTA, negative controls were performed on parallel sections. The reaction was halted by removing the substrate solution and incubating for 10 min in PBS containing 4% paraformaldehyde. The green fluorescence from gelatinase activity was then evaluated using a Leica ST5 laser-scanning confocal microscope as well as to collect immunofluorescence images. For the quantification of *in situ* zymography, Image J was used to compare the mean fluorescence intensity of the entire aorta.

### Aortic Ultrasonography Monitoring

We employed a 450 MHz transducer on a high-resolution micro-ultrasound system (Vevo 2100; VisualSonics, Canada) for ultrasonography. Mouse hair was shaved from the chest, and the mice were sedated with 1% isoflurane to do ultrasonic imaging. The mice were positioned in a supine position with the probe at the right margin of the sternum. The probe was positioned 45° from the chest walls of the mice. Images of ascending aortas, aortic arches, and branches were obtained. Longitudinal images of the aorta were obtained in B-mode, and the internal diameter of the aorta was measured.

### Gelatin Zymography

To detect gelatinase activity, gelatin zymography was used. Hep G2 cells were pretreated with crocin (50 M) in serum-free media for 36 h at 37°C. The conditioned media was collected and cleaned by centrifugation at 1,500 g for 4 min before being combined with 4 native-PAGE loading buffer (Solarbio, China) and electrophoresed on an 8 percent sodium dodecyl sulfate-polyacrylamide gel with 0.1 percent (w/v) gelatin. Following electrophoresis, the gel was washed three times in 2.5 percent Triton X-100 for 20 min each time, then incubated for 36 h at 37°C in developing buffer (50 mM Tris-HCl pH 7.5, 1 M ZnCl2, 5 mM CaCl2, and 1 percent Triton X-100). Finally, the gel was stained for 1 hin a 40 percent methanol, 10% acetic acid, and 0.1 percent (w/v) Coomassie Blue R-250 solution before being destained in a 4 percent methanol, 8% acetic acid solution. Transparent bars in a dark blue background indicated gelatinolytic activity.

### Cell Invasion Experiment

The Corning^®^ BioCoatTM Matrigel^®^ Invasion Chamber (Corning, USA) was used to examine the ability of cells to degrade the extracellular matrix. To begin, the upper chamber was filled with 200 μL of DMEM culture fluid containing SL4 cells, while the lower chamber was filled with 500 μL of DMEM containing 20% FBS. Plates were cultivated for 48 h at 37°C. The matrix on the bottom of the top chamber was gently swept away, as were cells that failed to pass the membrane surface. The membrane was preserved with paraformaldehyde for 10 min. The cells were labeled with DAPI for 5 min after drying. Finally, a Leica ST5 laser-scanning confocal microscope was used to observe cell invasion.

### Real-Time PCR

Hep G2 cells were treated with 50 μM GM6001 or 50 μM crocin for 36 h and then harvested as before, and RNA was extracted using TRIzol Reagent (Invitrogen, Carlsbad, CA, USA). A total of 2 μg of RNA was reverse-transcribed to cDNA using the GoScript Reverse Transcription System (Promega, Madison, WI, USA). Real-time PCR was performed using SYBR Green II (Takara, Shiga, Japan) for 35 cycles in an iQ5 system (BioRad, Hercules, CA, USA) using the primers below.

Primers were designed using the nucleotide database (NCBI). Primers were synthesized by SinoGenoMax, Beijing, China. The sequences of the primers used are as follows:

h-MMP2-F: CCCACTGCGGTTTTCTCGAAT

h-MMP2-R: CAAAGGGGTATCCATCGCCAT

h-TIMP2-F: GCTGCGAGTGCAAGATCAC

h-TIMP2-R: TGGTGCCCGTTGATGTTCTTC

h-GAPDH-F: AATGCATCCTGCACCACC

h-GAPDH-R: ATGCCAGTGAGCTTCCCG.

### Western Blotting

Hep G2 cells were treated with 50 μM GM6001 or 50 μM crocin for 36 h. After lysis with T-PER tissue protein extraction reagent containing protease/phosphatase inhibitors (Thermo Scientific, USA), total protein was recovered from an equivalent number of cells. Before being transferred to nitrocellulose membranes, proteins were separated on 8 percent sodium dodecyl sulfate-polyacrylamide electrophoresis gels (Millipore, USA). Membranes were blocked by TBST with 5% BSA (BD Bioscience, USA) for 1 hat 15–25°C. At 4°C overnight, the membranes were treated with the primary antibodies against MMP2 (ab37150, Abcam, UK) and GAPDH (ab8245, Abcam, UK). The secondary antibodies were then incubated for 1 hat 15–25°C with infrared dye 800-conjugated secondary antibodies (1: 10000, Rockland Immunochemicals, USA). The Odyssey infrared imaging equipment was used to quantify the images (LI-COR Biosciences, USA).

### Biolayer Interferometry

Biolayer interferometry was performed using an Octet RED384 instrument (ForteBio). Recombinant Human MMP2 was purchased from Novoprotein, China (P08253) and was reconstitution in distilled water with a concentration of 500 μg/ml. EZ-Link NHS-PeG4-Biotin (Thermo, part no. 21329) was prepared as a mother liquor with a concentration of 10 mM. MMP2 protein and biotin solution were mixed to a biotin: protein molar ratio of 1:1 and then reacted at 15–25°C for 30 min. Biotinylated MMP2 was put in a desalting column and centrifuged for 5 min at 1,500 rpm, and the flow-through protein was collected. Streptavidin biosensors were loaded with biotinylated MMP2, equilibrated in the solvent of crocin solution for 30 s, then dipped into crocin solution at varying concentrations until equilibrium was observed. Affinity (Kd) and kinetic parameters (kon and koff) were calculated from a global fit (1:1) of the data.

### Statistical Analysis

All statistical analyses were performed using GraphPad Prism 9.0 (San Diego, CA, USA). Data are expressed as the mean ± SEM. The unpaired two-tailed *t*-test was used to assess the differences between two groups. Fisher's exact test was used to assess the the differences of incidence rate or rupture rate between two groups. Log-rank (Mantel-Cox) test (conservative) was used to assess the differences of survival between two groups. Statistical significance was set at *P* < 0.05.

## Data Availability Statement

The original contributions presented in the study are included in the article/Supplementary Material, further inquiries can be directed to the corresponding author/s.

## Ethics Statement

The animal study was reviewed and approved by Institutional Animal Care and Use Committee of Capital Medical University.

## Author Contributions

JD and PL: conceptualization. FQ, YZ, HZ, and MZ: methodology. KZ and JC: software. KX: investigation. FQ and YL: data curation. FQ and SZ: visualization. FQ: writing—original draft. KZ, YL, PL, and JD: writing—review and editing. JD: supervision. All authors contributed to subsequent drafts and approved the submitted version.

## Funding

This project was supported by the National Natural Science Foundation of China (grant numbers 81930014, 81770250, 91939106, 81790622, and 81870339), Beijing Advanced Innovation Center for Big Data-based Precision Medicine, and Capital Medical University, Beijing (PXM2021_014226_000026).

## Conflict of Interest

The authors declare that the research was conducted in the absence of any commercial or financial relationships that could be construed as a potential conflict of interest.

## Publisher's Note

All claims expressed in this article are solely those of the authors and do not necessarily represent those of their affiliated organizations, or those of the publisher, the editors and the reviewers. Any product that may be evaluated in this article, or claim that may be made by its manufacturer, is not guaranteed or endorsed by the publisher.

## References

[1] Davies RR Goldstein LJ Coady MA Tittle SL Rizzo JA Kopf GS. Yearly rupture or dissection rates for thoracic aortic aneurysms: simple prediction based on size. Ann. Thorac. Surg. (2002) 73:17–27. 10.1016/S0003-4975(01)03236-2 11834007

[2] Mussa FF Horton JD Moridzadeh R Nicholson J Trimarchi S Eagle KA. Acute aortic dissection and intramural hematoma: a systematic review. JAMA. (2016) 316:754. 10.1001/jama.2016.10026 27533160

[3] Authors/Task Force m Erbel R Aboyans V Boileau C Bossone E Bartolomeo RD. ESC guidelines on the diagnosis and treatment of aortic diseases: document covering acute and chronic aortic diseases of the thoracic and abdominal aorta of the adult the task force for the diagnosis and treatment of aortic diseases of the European Society of Cardiology (ESC). Eur Heart J. (2014) 35:2873–926. 10.1093/eurheartj/ehu281 25173340

[4] Isselbacher EM Bonaca MP Di Eusanio M Froehlich J Bassone E Sechtem U. Recurrent aortic dissection: observations from the international registry of aortic dissection. Circulation. (2016) 134:1013–24. 10.1161/CIRCULATIONAHA.115.019359 27587434

[5] Milewicz DM Braverman AC De Backer J Morris SA Boileau C Maumenee IH. Marfan syndrome. Nat Rev Dis Primers. (2021) 7:64. 10.1038/s41572-021-00298-7 34475413 PMC9261969

[6] He R Guo DC Estrera AL Safi HJ Huynh TT Yin Z. Characterization of the inflammatory and apoptotic cells in the aortas of patients with ascending thoracic aortic aneurysms and dissections. J Thorac Cardiovasc Surg. (2006) 131:671–8. 10.1016/j.jtcvs.2005.09.018 16515922

[7] Wu D Shen YH Russell L Coselli JS LeMaire SA. Molecular mechanisms of thoracic aortic dissection. J Surg Res. (2013) 184:907–24. 10.1016/j.jss.2013.06.007 23856125 PMC3788606

[8] Ijaz T Tilton RG Brasier AR. Cytokine amplification and macrophage effector functions in aortic inflammation and abdominal aortic aneurysm formation. J Thorac Dis. (2016) 8:E746–E54. 10.21037/jtd.2016.06.37 27619163 PMC4999690

[9] Cifani N Proietta M Tritapepe L Di Gioia C Ferri L Taurino M. Stanford-A acute aortic dissection, inflammation, and metalloproteinases: a review. Ann Med. (2015) 47:441–6. 10.3109/07853890.2015.1073346 26339779

[10] Rabkin SW. The role matrix metalloproteinases in the production of aortic aneurysm. Prog Mol Biol Transl Sci. (2017) 147:239–65. 10.1016/bs.pmbts.2017.02.002 28413030

[11] Liu S Zhang X Wang J. Isovitexin protects against cisplatin-induced kidney injury in mice through inhibiting inflammatory and oxidative responses. Int Immunopharmacol. (2020) 83:106437. 10.1016/j.intimp.2020.106437 32222637

[12] Zhang Y Qi Z Wang W Wang L Cao F Zhao L. Isovitexin inhibits ginkgolic acids-induced inflammation through downregulating SHP2 activation. Front Pharmacol. (2021) 12:630320. 10.3389/fphar.2021.630320 34456714 PMC8385789

[13] Lv H Yu Z Zheng Y Wang L Qin X Cheng G. Isovitexin exerts anti-inflammatory and anti-oxidant activities on lipopolysaccharide-induced acute lung injury by inhibiting MAPK and NF-κB and activating HO-1/Nrf2 pathways. Int J Biol Sci. (2016) 12:72–86. 10.7150/ijbs.13188 26722219 PMC4679400

[14] Ikram M Muhammad T Rehman SU Khan A Jo MG Ali T. Hesperetin confers neuroprotection by regulating Nrf2/TLR4/NF-κB signaling in an Aβ mouse model. Mol Neurobiol. (2019) 56:6293–309. 10.1007/s12035-019-1512-7 30756299

[15] Lin Z Fu C Yan Z Wu Y Zhan J Lou Z. The protective effect of hesperetin in osteoarthritis: an *in vitro* and *in vivo* study. Food Funct. (2020) 11:2654–66. 10.1039/C9FO02552A 32159191

[16] Chen X Wei W Li Y Huang J Ci X. Hesperetin relieves cisplatin-induced acute kidney injury by mitigating oxidative stress, inflammation and apoptosis. Chem Biol Interact. (2019) 308:269–78. 10.1016/j.cbi.2019.05.040 31153982

[17] Godugu C Pasari LP Khurana A Anchi P Saifi MA Bansod SP. Crocin, an active constituent of crocus sativus ameliorates cerulein induced pancreatic inflammation and oxidative stress. Phytother Res. (2020) 34:825–35. 10.1002/ptr.6564 31769107

[18] Ben Salem I Boussabbeh M Helali S Abid-Essefi S Bacha H. Protective effect of crocin against zearalenone-induced oxidative stress in liver and kidney of Balb/c mice. Environ Sci Pollut Res Int. (2015) 22:19069–76. 10.1007/s11356-015-5086-2 26233739

[19] Boussabbeh M Ben Salem I Neffati F Najjar MF Bacha H Abid-Essefi S. Crocin prevents patulin-induced acute toxicity in cardiac tissues via the regulation of oxidative damage and apoptosis. J Biochem Mol Toxicol. (2015) 29:479–88. 10.1002/jbt.21718 26095701

[20] Ren W Liu Y Wang X Jia L Piao C Lan F. β-Aminopropionitrile monofumarate induces thoracic aortic dissection in C57BL/6 mice. Sci Rep. (2016) 6:28149. 10.1038/srep28149 27329825 PMC4916438

[21] Xu K Xu C Zhang Y Qi F Yu B Li P. Identification of type IV collagen exposure as a molecular imaging target for early detection of thoracic aortic dissection. Theranostics. (2018) 8:437–49. 10.7150/thno.22467 29290819 PMC5743559

[22] Diaz-Canestro C Puspitasari YM Liberale L Guzik TJ Flammer AJ Bonetti NR. MMP-2 knockdown blunts age-dependent carotid stiffness by decreasing elastin degradation and augmenting eNOS activation. Cardiovasc Res. (2021) cvab300. 10.1093/cvr/cvab300 34586381

[23] Jumper J Evans R Pritzel A Green T Figurnov M Ronneberger O. Highly accurate protein structure prediction with AlphaFold. Nature. (2021) 596:583–9. 10.1038/s41586-021-03819-2 34265844 PMC8371605

[24] Lindsay ME Dietz HC. Lessons on the pathogenesis of aneurysm from heritable conditions. Nature. (2011) 473:308–16. 10.1038/nature10145 21593863 PMC3622871

[25] Hobeika MJ Thompson RW Muhs BE Brooks PC Gagne PJ. Matrix metalloproteinases in peripheral vascular disease. J Vasc Surg. (2007) 45:849–57. 10.1016/j.jvs.2006.09.066 17398401

[26] Ishii T Asuwa N. Collagen and elastin degradation by matrix metalloproteinases and tissue inhibitors of matrix metalloproteinase in aortic dissection. Hum Pathol. (2000) 31:640–6. 10.1053/hupa.2000.7642 10872655

[27] Giachino F Loiacono M Lucchiari M Manzo M Battista S Saglio E. Rule out of acute aortic dissection with plasma matrix metalloproteinase 8 in the emergency department. Crit Care. (2013) 17:R33. 10.1186/cc12536 23442769 PMC4057269

[28] Karapanagiotidis GT Antonitsis P Charokopos N Foroulis CN Anastasiadis K Rouska E. Serum levels of matrix metalloproteinases−1,-2,-3 and−9 in thoracic aortic diseases and acute myocardial ischemia. J Cardiothorac Surg. (2009) 4:59. 10.1186/1749-8090-4-59 19886986 PMC2774681

[29] Ghosh A Pechota A Coleman D Upchurch GR. Jr., Eliason JL. Cigarette smoke-induced MMP2 and MMP9 secretion from aortic vascular smooth cells is mediated via the Jak/Stat *pathway. Hum Pathol*. (2015) 46:284–94. 10.1016/j.humpath.2014.11.003 25537973

[30] Longo GM Xiong W Greiner TC Zhao Y Fiotti N Baxter BT. Matrix metalloproteinases 2 and 9 work in concert to produce aortic aneurysms. J Clin Invest. (2002) 110:625–32. 10.1172/JCI0215334 12208863 PMC151106

[31] Zhang X Wu D Choi JC Minard CG Hou X Coselli JS. Matrix metalloproteinase levels in chronic thoracic aortic dissection. J Surg Res. (2014) 189:348–58. 10.1016/j.jss.2014.03.027 24746253 PMC4065027

[32] Robbins CS Byrne JS. Interleukin-3 is required for thoracic aneurysm and dissection in a mouse model. Clin Sci. (2018) 132:1253–6. 10.1042/CS20180185 29930143

[33] Chung AW Yang HH Radomski MW van Breemen C. Long-term doxycycline is more effective than atenolol to prevent thoracic aortic aneurysm in marfan syndrome through the inhibition of matrix metalloproteinase-2 and−9. Circ Res. (2008) 102:e73–85. 10.1161/CIRCRESAHA.108.174367 18388324

[34] Baxter BT Matsumura J Curci JA McBride R Larson L Blackwelder W. Effect of doxycycline on aneurysm growth among patients with small infrarenal abdominal aortic aneurysms: a randomized clinical trial. Jama. (2020) 323:2029–38. 10.1001/jama.2020.5230 32453369 PMC7251450

[35] Newman DJ Cragg GM. Natural products as sources of new drugs from 1981 to 2014. J Nat Prod. (2016) 79:629–61. 10.1021/acs.jnatprod.5b01055 26852623

[36] Hibender S Franken R van Roomen C Ter Braake A van der Made I Schermer EE. Resveratrol inhibits aortic root dilatation in the Fbn1C1039G/+ marfan mouse model. Arterioscler Thromb Vasc Biol. (2016) 36:1618–26. 10.1161/ATVBAHA.116.307841 27283746 PMC4961273

[37] Setozaki S Minakata K Masumoto H Hirao S Yamazaki K Kuwahara K. Prevention of abdominal aortic aneurysm progression by oral administration of green tea polyphenol in a rat model. Eur J Vasc Surg. (2017) 65:1803–12.e2. 10.1016/j.jvs.2016.06.003 27473778

[38] Alavizadeh SH Hosseinzadeh H. Bioactivity assessment and toxicity of crocin: a comprehensive review. Food Chem Toxicol. (2014) 64:65–80. 10.1016/j.fct.2013.11.016 24275090

[39] Mokhtari-Zaer A Khazdair MR Boskabady MH. Smooth muscle relaxant activity of crocus sativus (saffron) and its constituents: possible mechanisms. Avicenna J Phytomedicine. (2015) 5:365–75. 26468456 PMC4599118

[40] He SY Qian ZY Tang FT. Effect of crocin on intracellular calcium concentration in cultured bovine aortic smooth muscle cells. Yao Xue Xue Bao. (2004) 39:778–81. 15700815

[41] Imenshahidi M Razavi BM Faal A Gholampoor A Mousavi SM Hosseinzadeh H. Effects of chronic crocin treatment on desoxycorticosterone acetate (doca)-salt hypertensive rats. Iran J Basic Med Sci. (2014) 17:9–13. 24592301 PMC3938880

[42] He SY Qian ZY Tang FT Wen N Xu GL Sheng L. Effect of crocin on experimental atherosclerosis in quails and its mechanisms. Life Sci. (2005) 77:907–21. 10.1016/j.lfs.2005.02.006 15964309

[43] Li K Li Y Ma Z Zhao J. Crocin exerts anti-inflammatory and anti-catabolic effects on rat intervertebral discs by suppressing the activation of JNK. Int J Mol Med. (2015) 36:1291–9. 10.3892/ijmm.2015.2359 26648423 PMC4601741

[44] Piñero J Ramírez-Anguita JM Saüch-Pitarch J Ronzano F Centeno E Sanz F. The DisGeNET knowledge platform for disease genomics: 2019 update. Nucleic Acids Res. (2020) 48:D845–55. 10.1093/nar/gkz1021 31680165 PMC7145631

[45] Jassal B Matthews L Viteri G Gong C Lorente P Fabregat A. The reactome pathway knowledgebase. Nucleic Acids Res. (2020) 48:D498–503. 10.1093/nar/gkz1031 31691815 PMC7145712

[46] Burley SK Berman HM Bhikadiya C Bi C Chen L Di Costanzo L. RCSB Protein Data Bank: biological macromolecular structures enabling research and education in fundamental biology, biomedicine, biotechnology and energy. Nucleic Acids Res. (2019) 47:D464–74. 10.1093/nar/gky1004 30357411 PMC6324064

[47] Cabarcas-Montalvo M Maldonado-Rojas W Montes-Grajales D Bertel-Sevilla A Wagner-Döbler I Sztajer H. Discovery of antiviral molecules for dengue: in silico search and biological evaluation. Eur J Med Chem. (2016) 110:87–97. 10.1016/j.ejmech.2015.12.030 26807547

[48] Wright B Watson KA McGuffin LJ Lovegrove JA Gibbins JM. GRID and docking analyses reveal a molecular basis for flavonoid inhibition of Src family kinase activity. J Nutr Biochem. (2015) 26:1156–65. 10.1016/j.jnutbio.2015.05.004 26140983

[49] Jain AN. Surflex: fully automatic flexible molecular docking using a molecular similarity-based search engine. J Med Chem. (2003) 46:499–511. 10.1021/jm020406h 12570372

[50] Orabi KY Abaza MS El Sayed KA Elnagar AY Al-Attiyah R Guleri RP. Selective growth inhibition of human malignant melanoma cells by syringic acid-derived proteasome inhibitors. Cancer Cell Int. (2013) 13:82. 10.1186/1475-2867-13-82 23958424 PMC3765228

[51] Liu W Meng Q Sun Y Wang C Huo X Liu Z. Targeting P-glycoprotein: nelfinavir reverses adriamycin resistance in K562/ADR cells. Cell Physiol Biochem. (2018) 51:1616–31. 10.1159/000495650 30497065

[52] Fashandi AZ Spinosa M Salmon M Su G Montgomery W Mast A. Female mice exhibit abdominal aortic aneurysm protection in an established rupture model. J Surg Res. (2020) 247:387–96. 10.1016/j.jss.2019.10.004 31699539 PMC7111562

[53] Qi X Wang F Chun C Saldarriaga L Jiang Z Pruitt EY. A validated mouse model capable of recapitulating the protective effects of female sex hormones on ascending aortic aneurysms and dissections (AADs). Physiol Rep. (2020) 8:e14631. 10.14814/phy2.14631 33242364 PMC7690909

[54] Zhang C van der Voort D Shi H Zhang R Qing Y Hiraoka S. Matricellular protein CCN3 mitigates abdominal aortic aneurysm. J Clin Invest. (2016) 126:1282–99. 10.1172/JCI82337 26974158 PMC4811126

[55] Tang EH Shvartz E Shimizu K Rocha VZ Zheng C Fukuda D. Deletion of EP4 on bone marrow-derived cells enhances inflammation and angiotensin II-induced abdominal aortic aneurysm formation. Arterioscler Thromb Vasc Biol. (2011) 31:261–9. 10.1161/ATVBAHA.110.216580 21088251 PMC3025710

[56] Gonzalez JM Franzke CW Yang F Romero R Girardi G. Complement activation triggers metalloproteinases release inducing cervical remodeling and preterm birth in mice. Am J Pathol. (2011) 179:838–49. 10.1016/j.ajpath.2011.04.024 21801872 PMC3157168

