# Supplementary material for: Artificial Intelligence Uncovers Natural MMP Inhibitor Crocin as a Potential Treatment of Thoracic Aortic Aneurysm and Dissection
Source: Front Cardiovasc Med. 2022 Apr 6;9:871486. doi: 10.3389/fcvm.2022.871486 (PMC9019136; doi:10.3389/fcvm.2022.871486)
Supplement: Supplementary file 1 [file Data_Sheet_1.pdf]

Supplementary Material

1. Supplementary Data

Supplementary Table 1. 41 TAAD-associated genes of "Aortic Aneurysm, Thoracic, C0162872" in DisGeNET database

No.	Gene	Gene_id	UniProt	Gene_Full_Name	Score_gda
1	ACR	49	P10323	acrosin	0.01
2	ACTA2	59	P62736	actin alpha 2, smooth muscle	0.43
3	ACTB	60	P60709	actin beta	0.02
4	ADAM10	102	O14672	ADAM metallopeptidase domain 10	0.2
5	ADAM17	6868	P78536	ADAM metallopeptidase domain 17	0.21
6	AGT	183	P01019	angiotensinogen	0.02
7	ANKRD44-IT1	101927547		ANKRD44 intronic transcript 1	0.1
8	ANKRD44	91526	Q8N8A2	ankyrin repeat domain 44	0.1
9	BGN	633	P21810	biglycan	0.01
10	CDKN2B-AS1	100048912		CDKN2B antisense RNA 1	0.1
11	ELN	2006	P15502	elastin	0.02
12	FBN1	2200	P35555	fibrillin 1	0.46
13	FLNA	2316	P21333	filamin A	0.01
14	HOTAIR	100124700		HOX transcript antisense RNA	0.01
15	IL1B	3553	P01584	interleukin 1 beta	0.01
16	IL6	3569	P05231	interleukin 6	0.01
17	KLF4	9314	O43474	Kruppel like factor 4	0.01
18	LTBP3	4054	Q9NS15	latent transforming growth factor beta binding protein 3	0.01
19	LOX	4015	P28300	lysyl oxidase	0.02
20	MMP2	4313	P08253	matrix metallopeptidase 2	0.52
21	MMP9	4318	P14780	matrix metallopeptidase 9	0.51

22	MFAP5	8076	Q13361	microfibril associated protein 5	0.1
23	MIR21	406991		microRNA 21	0.01
24	MIR574	693159		microRNA 574	0.01
25	MYH11	4629	P35749	myosin heavy chain 11	0.03
26	MYLK	4638	Q15746	myosin light chain kinase	0.01
27	NOTCH1	4851	P46531	notch receptor 1	0.01
28	PRKCB	5579	P05771	protein kinase C beta	0.01
29	PRKG1	5592	Q13976	protein kinase cGMP-dependent 1	0.11
30	ARHGAP18	93663	Q8N392	Rho GTPase activating protein 18	0.01
31	ROBO4	54538	Q8WZ75	roundabout guidance receptor 4	0.3
32	S100A12	6283	P80511	S100 calcium binding protein A12	0.01
33	SERPINE1	5054	P05121	serpin family E member 1	0.01
34	SERPINE2	5270	P07093	serpin family E member 2	0.01
35	SMAD3	4088	P84022	SMAD family member 3	0.01
36	SMAD4	4089	Q13485	SMAD family member 4	0.02
37	THBS1	7057	P07996	thrombospondin 1	0.01
38	TGFB2	7042	P61812	transforming growth factor beta 2	0.31
39	TGFBR2	7048	P37173	transforming growth factor beta receptor 2	0.33
40	TNF	7124	P01375	tumor necrosis factor	0.01
41	VEGFA	7422	P15692	vascular endothelial growth factor A	0.01

Supplementary Table 2. 57 TAAD-associated genes of "Dissecting aneurysm of the thoracic aorta, C0729233" in DisGeNET database

No.	Gene	Gene_id	UniProt	Gene_Full_Name	Score_gda
1	IS1	260402		Adolescent idiopathic scoliosis	0.01
2	ALK	238	Q9UM73	ALK receptor tyrosine kinase	0.01
3	AR	367	P10275	androgen receptor	0.01
4	AGT	183	P01019	angiotensinogen	0.01
5	APOE	348	P02649	apolipoprotein E	0.02
6	BEST1	7439	O76090	bestrophin 1	0.01
7	CALCR	799	P30988	calcitonin receptor	0.01
8	CALR	811	P27797	calreticulin	0.01
9	CA2	760	P00918	carbonic anhydrase 2	0.01
10	CTSK	1513	P43235	cathepsin K	0.01
11	CTSL	1514	P07711	cathepsin L	0.01
12	CCL3	6348	P10147	C-C motif chemokine ligand 3	0.01
13	CD274	29126	Q9NZQ7	CD274 molecule	0.01
14	CD38	952	P28907	CD38 molecule	0.03
15	CD6	923	P30203	CD6 molecule	0.03
16	CST3	1471	P01034	cystatin C	0.01
17	DAO	1610	P14920	D-amino acid oxidase	0.01
18	DMD	1756	P11532	dystrophin	0.01
19	EFEMP1	2202	Q12805	EGF containing fibulin extracellular matrix protein 1	0.02
20	ELN	2006	P15502	elastin	0.01
21	EGFR	1956	P00533	epidermal growth factor receptor	0.02
22	FBN1	2200	P35555	fibrillin 1	0.01
23	FHIT	2272	P49789	fragile histidine triad diadenosine triphosphatase	0.01
24	GLA	2717	P06280	galactosidase alpha	0.01
25	ICAM1	3383	P05362	intercellular adhesion molecule 1	0.01

26	IL1B	3553	P01584	interleukin 1 beta	0.01
27	IL10	3586	P22301	interleukin 10	0.01
28	IL33	90865	O95760	interleukin 33	0.01
29	LINC01194	404663		long intergenic non-protein coding RNA 1194	0.01
30	MAEL	84944	Q96JY0	maelstrom spermatogenic transposon silencer	0.01
31	MMP2	4313	P08253	matrix metallopeptidase 2	0.01
32	MMP9	4318	P14780	matrix metallopeptidase 9	0.02
33	MTOR	2475	P42345	mechanistic target of rapamycin kinase	0.01
34	MIR146A	406938		microRNA 146a	0.01
35	MIR155	406947		microRNA 155	0.01
36	MIR210	406992		microRNA 210	0.01
37	MYCN	4613	P04198	MYCN proto-oncogene, bHLH transcription factor	0.01
38	NOS3	4846	P29474	nitric oxide synthase 3	0.01
39	PLAT	5327	P00750	plasminogen activator, tissue type	0.01
40	PFN1	5216	P07737	profilin 1	0.01
41	SPINK4	27290	O60575	serine peptidase inhibitor Kazal type 4	0.01
42	SMUG1	23583	Q53HV7	single-strand-selective monofunctional uracil-DNA glycosylase 1	0.03
43	SLC25A20	788	O43772	solute carrier family 25 member 20	0.01
44	SLC39A13	91252	Q96H72	solute carrier family 39 member 13	0.01
45	SLC6A8	6535	P48029	solute carrier family 6 member 8	0.01
46	STS	412	P08842	steroid sulfatase	0.01
47	SDHD	6392	O14521	succinate dehydrogenase complex subunit D	0.01
48	SMN1	6606	Q16637	survival of motor neuron 1, telomeric	0.01
49	SMN2	6607	Q16637	survival of motor neuron 2, centromeric	0.01
50	TIMP3	7078	P35625	TIMP metallopeptidase inhibitor 3	0.01
51	TRAP1	10131	Q12931	TNF receptor associated protein 1	0.01
52	TGFB3	7043	P10600	transforming growth factor beta 3	0.01
53	TP53	7157	P04637	tumor protein p53	0.01

54	TP63	8626	Q9H3D4	tumor protein p63	0.01
55	VEGFA	7422	P15692	vascular endothelial growth factor A	0.01
56	WNT5A	7474	P41221	Wnt family member 5A	0.01
57	ZIC3	7547	O60481	Zic family member 3	0.01

Supplementary Table 3. Top 10 pathways of 147 pathways from TAAD-associated gene set analysis

The detailed results of 147 pathways are presented in the additional file (Supplementary File 1). 147 pathways are selected with Entities *P* value< 0.05. Top 10 pathways were ranked by Entities *P* value.

**Pathway identifier**	**Pathway name**	**Entities P value**	**Entities FDR**	**Submitted entities found**
**R-HSA-1474244**	Extracellular matrix organization	4.91E-14	3.20E-11	TGFB2;TGFB3;MMP2;ELN;SERPINE1;BGN;ADAM10;PLAT;LTBP3;MMP9;THBS1;ICAM1;MFAP5;ADAM17;EFEMP1;LOX;CTSL;CTSK;DMD;FBN1
**R-HSA-6785807**	Interleukin-4 and Interleukin-13 signaling	1.22E-13	3.99E-11	IL10;IL6;MMP2;IL1B;TP53;TNF;MMP9;VEGFA;ICAM1
**R-HSA-1566948**	Elastic fibre formation	2.45E-11	5.32E-09	MFAP5;TGFB2;EFEMP1;LOX;TGFB3;ELN;LTBP3;FBN1
**R-HSA-6783783**	Interleukin-10 signaling	3.75E-11	6.12E-09	IL10;IL6;IL1B;CCL3;TNF;ICAM1
**R-HSA-2129379**	Molecules associated with elastic fibres	1.19E-10	1.54E-08	MFAP5;TGFB2;EFEMP1;TGFB3;ELN;LTBP3;FBN1
**R-HSA-449147**	Signaling by Interleukins	9.80E-10	1.06E-07	IL10;IL33;SMAD3;MMP2;TNF;MMP9;VEGFA;ICAM1;IL6;CA2;IL1B;CCL3;S100A12;TP53
**R-HSA-9006934**	Signaling by Receptor Tyrosine Kinases	5.36E-08	4.98E-06	ALK;CD274;NOS3;PRKCB;ADAM10;PLAT;MMP9;THBS1;MTOR;EGFR;ACTB;VEGFA;ADAM17;MYCN;APOE
**R-HSA-1442490**	Collagen degradation	3.05E-07	2.47E-05	ADAM17;CTSL;CTSK;MMP2;ADAM10;PLAT;MMP9
**R-HSA-1280215**	Cytokine Signaling in Immune system	6.04E-07	4.35E-05	IL10;IL33;SMAD3;MMP2;MMP9;TNF;MTOR;VEGFA;ICAM1;IL6;ADAM17;CA2;IL1B;CCL3;FLNA;S100A12;TP53
**R-HSA-76005**	Response to elevated platelet cytosolic Ca2+	1.33E-06	7.83E-05	TGFB2;TGFB3;PRKCB;SERPINE1;FLNA;TIMP3;PFN1;THBS1;VEGFA

Supplementary Table 4. 20 TAAD-related genes in "Extracellular matrix organization".

Genes were ranked by total GDA score.

Gene	GDA score of C0162872	GDA score of C0729233	Total GDA score
MMP2	0.52	0.01	0.53
MMP9	0.51	0.02	0.53
FBN1	0.01	0.46	0.47
TGFB2	0.31		0.31
ADAM17	0.21		0.21
ADAM10	0.2		0.2
MFAP5	0.1		0.1
EFEMP1	0.02		0.02
LOX	0.02		0.02
BGN	0.01		0.01
CTSK	0.01		0.01
CTSL	0.01		0.01
DMD	0.01		0.01
ICAM1	0.01		0.01
LTBP3	0.01		0.01
SERPINE1	0.01		0.01
TGFB3	0.01		0.01
THBS1	0.01		0.01

Supplementary Table 5. Predictive binding targets for 9 compounds in Virtual screening.

Mol_ID	Compound	Targets
MOL000006	Luteolin	MMP1 MMP8 MMP10 MMP16
MOL001406	Crocin	MMP1 MMP2 MMP3 MMP10 MMP14
MOL001445	Leonurine	MMP2 MMP7 MMP8 MMP10 MMP16
MOL001689	Acacetin	MMP1 MMP3 MMP10 MMP14
MOL002051	Isoquercitrin	MMP8 MMP12 MMP16 MMP20
MOL002322	Isovitexin	MMP1 MMP8 MMP10
MOL002341	Hesperetin	MMP8 MMP9 MMP14
MOL002931	Scutellarin	MMP2 MMP3 MMP8 MMP10
MOL013077	Decursin	MMP1 MMP8 MMP16 MMP20

1. Supplementary Figures

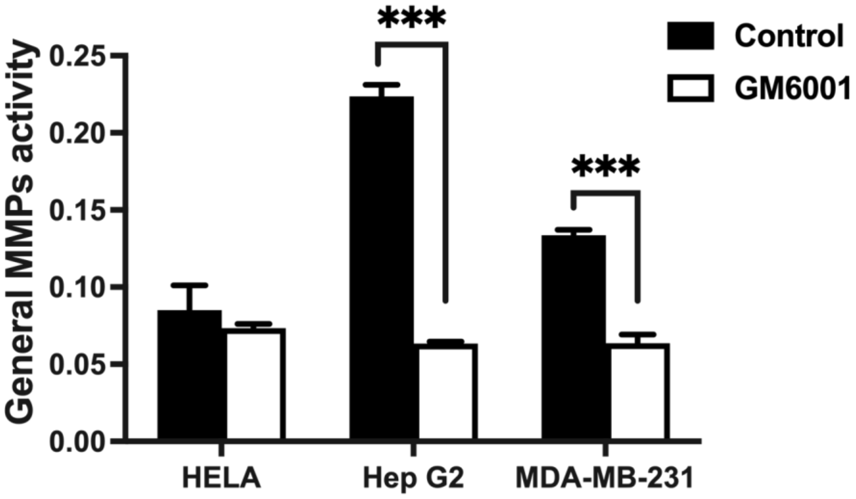

Supplementary Figure 2. General MMP activity on HELA, Hep G2, and MDA-MB-231 cells.

HELA, Hep G2, and MDA-MB-231 cells were incubated for 36 h after treated with 50 µM GM6001, and the total MMPs activity in cell supernatant was measured by general MMP activity kit. n = 3, values were expressed as the mean ± SD, and analyzed by unpaired t test. ***p < 0.001 vs control group.
